# Associations between Exposure to Tobacco Smoke and Behavioral Problems in Preschool Japanese Children

**DOI:** 10.1155/2020/7591263

**Published:** 2020-05-14

**Authors:** Keiko Wada, Jun Ueyama, Kie Konishi, Yuko Goto, Sachi Koda, Fumi Mizuta, Takashi Tamura, Kaori Watanabe, Kyoko Ando, Takaaki Kondo, Chisato Nagata

**Affiliations:** ^1^Department of Epidemiology and Preventive Medicine, Gifu University Graduate School of Medicine, Gifu, Japan; ^2^Department of Pathophysiological Laboratory Sciences, Field of Radiological and Medical Laboratory Sciences, Nagoya University Graduate School of Medicine, Nagoya, Japan; ^3^Department of Preventive Medicine, Nagoya University Graduate School of Medicine, Nagoya, Japan; ^4^Department of Food and Culture Science, Aichi Bunkyo Women's College, Inazawa, Japan

## Abstract

**Background:**

A few studies related to pediatric behavior have measured secondhand smoke exposure in children using valid objective biochemical markers. We aimed at investigating the associations between current and cumulative exposure to tobacco smoke, measured both subjectively and objectively, and behavioral problems in children.

**Methods:**

Subjects were 437 Japanese children, aged 3–6 years in 2006. Exposure to tobacco smoke was evaluated from a parent-administered questionnaire and urinary cotinine concentrations. The cotinine concentrations were measured using first-void morning urine by liquid chromatography-tandem mass spectrometry. Children's behaviors were assessed by the parent-completed Strengths and Difficulties Questionnaire.

**Results:**

After multiple adjustments for covariates, higher total difficulty scores of children were significantly associated with the larger number of cigarettes parents smoke, more smokers among cohabiters, and more pack-years of exposure to tobacco smoke from parents and cohabiters. The total difficulty scores were 8.72, 9.09, and 10.52, respectively, for children in the low, middle, and high tertiles of creatinine-corrected cotinine concentrations in urine (*p*=0.002, trend *p*=0.005). There was no substantial sex difference in the positive associations between passive smoking and the SDQ scores.

**Conclusions:**

Exposure to tobacco smoke in early childhood may be involved in the development of pediatric behavioral problems. The importance of reducing the exposure of children to tobacco smoke, particularly in the home, was further emphasized for the prevention of psychological and behavioral problems in childhood.

## 1. Introduction

In Japan, about 32% of men and 8% of women are smokers today [1]. Recently, exposure to tobacco smoke in public places has been decreasing under government regulation, whereas many people are still exposed to secondhand smoke (SHS) at home [1]. About half of Japanese children are estimated to live with one or more smokers at home [2–5]. Since childhood is a period when various organs develop rapidly with physical growth, exposure to tobacco smoke may have a harmful effect on child health. Previous studies have reported the association between SHS exposure in childhood and elevated rates of lower respiratory tract infections, asthma, dental caries, and otitis media [4–10].

Recently, a growing body of evidence has indicated that SHS exposure may influence mental and behavioral health in children [9–21]. Many cross-sectional [15–21] and longitudinal [12] studies have reported positive associations between SHS exposure during childhood and behavior problems including attention-deficit hyperactivity disorder, conduct disorder, and disruptive behavior, although some longitudinal studies did not observe significant association [13, 14]. So far, most of the previous studies have assessed SHS exposure using only the questionnaire [12–16], mainly based on parent's smoking habits. However, there are a few studies that have measured SHS exposure using objective biomarkers [17–21]. The amount of toxic chemicals in tobacco smoke taken into the body may vary due to environmental factors such as time spent with smokers and house size [22]. Biomarkers such as nicotine or cotinine can reflect tobacco smoke from a variety of sources, including parents, cohabiters, and others in public spaces. Also, since children might have been exposed to tobacco smoke for many years after birth [22], cumulative SHS exposure should be assessed comprehensively.

In the present study, we purposed to evaluate the associations between exposure to SHS and behavioral problems in young Japanese children. Not only we assessed current and cumulative SHS exposure using a detailed questionnaire about parental and cohabiters' smoking, but also we measured urinary concentrations of total cotinine in children.

## 2. Methods

### 2.1. Subjects and Design

This study was administered in two preschools in Aichi Prefecture, Japan. The details of the study have been previously described elsewhere [23]. Subjects were children aged three to six years who attend one of the preschools. During October and November 2006, children's and parents' characteristics and lifestyles were obtained through a parent-administered questionnaire. Parents were also asked to provide their children's first-void morning urine. Among 533 preschool children, 459 (86.1%) participated in the study, with the written consent of a parent. The study protocol and informed consent procedure were approved by the Ethical Board of the Gifu University Graduate School of Medicine, Gifu, Japan.

### 2.2. Assessment of Exposure to Smoke

In the questionnaire, the parents were asked about their smoking habits: never, former, or current smokers. Smokers were defined as people who had smoked a total of at least 20 packs of cigarettes in their life. For former and current smokers, the number of cigarettes per day and the age of initiation into smoking were inquired. The age at smoking cessation was also inquired for former smokers. Similar inquiries were made as to family members who lived with the enrolled child. No information on the brand of cigarettes was collected.

The sum of cigarettes parents currently smoked (cigarettes per day) and the number of current smokers among cohabiters (persons) were calculated as indices of current exposure to SHS. Next, to measure cumulative exposure to SHS, the durations the child had been exposed to household smoking from birth until the time of data collection were calculated using the period that parents and cohabiters had smoked and the child's birthday. The sum of the products of the duration and the amount that parents or cohabiters had smoked was expressed as pack-years of exposure to SHS after birth. Subjects with no exposure were assigned as zero pack-years. The residual subjects were divided into half (low or high).

Urine first voided after a child woke up was collected at home. Parents reported the time when the child urinated in the morning. The urines were brought to the kindergartens at 9-10 a.m. and then immediately transported to our laboratory. Preservative additives were not used. Immediately after arrival in the laboratory, urine samples were aliquoted and kept frozen at −80°C until the measurement. We measured urinary concentrations of total cotinine, a metabolite of nicotine, which can provide a biological estimate of exposure to SHS among nonsmokers [24]. Cotinine's half-life (average: 16 hours) is longer than nicotine's (2 hours), and cotinine concentrations in urine are higher than those in other biological fluids such as blood and saliva [25]. Total (free plus conjugated) cotinine was reported to be highly correlated with free cotinine (*R* = 0.94) and the total concentrations were about 2.4-fold higher relative to the free values in Japanese, resulting to decrease samples below the limit of detection (LOD) [24].

The total cotinine concentration (free cotinine plus its glucuronide) was measured by solid-phase extraction (SPE) procedure and liquid chromatography-tandem mass spectrometry (LC-MS/MS) detection after deconjugation reaction using *β*-glucuronidase obtained from Sigma-Aldrich (St. Louis, MO, USA). A polymeric SPE product, Bond Elut Plexa (30 mg) (Agilent Technologies, Inc., Santa Clara, CA, USA), was used for urinary cotinine extraction. LC-MS/MS analysis was run on an Agilent 1200 Infinity LC coupled with an Agilent 6430 Triple Quadrupole LC/MS System (Agilent Technologies, Inc., Santa Clara, CA, USA). Isotope-labeled cotinine (1.0 mg/ml methanol, cotinine-d3) was used as an internal standard. The within-run precision of our method was examined through the assay of pooled urine spiked with cotinine at concentrations of 0.4, 1.7, and 10 *μ*g/L (*n* = 4-5), and the results were less than 14.4% (relative standard deviation, %RSD). The accuracy of this method was certified by participation in the international program of the German External Quality Assessment Scheme (G-EQUAS). For 16 subjects (3.7%) with urinary cotinine concentrations under the detectable value (0.1 ng/ml), we compensated for their values by substituting the values of LOD/2. Urinary creatinine was measured by the conventional enzymatic method at SRL, Inc. (Tokyo, Japan). To adjust for variation in the dilution of urine, the creatinine-corrected concentration was calculated as the ratio of cotinine concentrations to creatinine concentrations (ng/mg creatinine). Since the distribution of creatinine-corrected cotinine concentrations was skewed, logarithmically transformed values were used in analyses. Urinary creatinine-corrected cotinine concentrations were also divided into three groups according to the tertiles (low, middle, or high).

### 2.3. Behavioral Problems

Children's behavior was assessed with the Japanese version of the Strengths and Difficulties Questionnaire (SDQ) developed by Goodman in 1997 [26], which is a brief instrument for assessing positive and negative aspects of the behavior of children and has twenty-five items divided into five subscales: emotional symptoms, conduct problems, hyperactivity, peer problems, and prosocial behaviors. SDQ is generally used as a screening tool for behavior problem in children. Each subscale has scores of 0–10. The total difficulty scores were calculated as the aggregate score of emotional symptoms, conduct problems, hyperactivity, and peer problems (the range: 0–40). The Japanese version of SDQ has been reported to be reliable [27]. In line with previous reports [26, 27], children with the upper 20% of total difficulty score were assigned to out of normal range (having behavior problems). As the results, the cutoff of the total difficulty score was set as 14.

### 2.4. Questionnaire on Potential Confounders

Mother's depression was assessed by the Center for Epidemiologic Studies Depression scale developed by Radloff [28]. A score of 16 or more was defined as depression. Children's heights, weights, and birthweights were reported by their parents in the questionnaire.

### 2.5. Statistical Analysis

The analyses were conducted among 437 children whose parents had responded regarding the smoking status of family members and the SDQ and provided their first-void morning urines for cotinine measurements. There were no significant differences in characteristics between participants analyzed and excluded.

The SDQ scores were compared according to the following five indices of SHS exposures by general linear models: (1) The sum of cigarettes parents currently smoked (0, >0 to 20, and 21 cigarettes per day or more). Each group was replaced with 0, 10, and 21 in the models to test a linear trend (trend *p*). (2) The number of current smokers among cohabiters (0, 1, and 2 persons or more). Tests for a linear trend were performed using 0, 1, and 2, respectively, in the models. (3) The pack-years of exposure to parents' smoking (0, low, or high) and (4) those to household smoking (0, low, or high). Tests for a linear trend were performed using the median values of each category. (5) The tertiles (low, middle, or high) of urinary creatinine-corrected cotinine concentrations. The continuous values were used in the model to test a linear trend (*p* for trend).

In addition, the odds ratios (ORs) and 95% confidence intervals (CIs) of behavioral problems were estimated according to each index of SHS exposures using the logistic regression model. Linear trends were tested as described above (*p* for trend).

Each analysis was performed after adjustments for the rater (mother, father), sex, age, body mass index, siblings (none, 1st, 2nd, or later), birthweight (g), feeding until 3 months old (breastfeeding only, mixed feeding, and bottle-feeding), age of mother at delivery (years old), mother's education (12 years or less, 13 to 15 years, and 16 years or more), and mother's depression (yes, no). Indicator terms were specifically created for missing data of categorical covariates.

All analyses were conducted using the SAS computer program, version 9.4 (SAS Institute). All *p* values were calculated using a two-sided test. For all analyses, a *p* value of less than 0.05 was inferred as statistically significant.

## 3. Results

Subjects were 231 boys and 206 girls with the mean age of 5.12 years. The SDQs were completed by mother (96.8%) or by father (3.2%). The mean (range) of total difficulty score was 9.43 (0–26). Urinary cotinine concentration ranged from 0.03 to 39.7 ng/mg creatinine, and the median was 1.41 ng/mg creatinine. The mean (95% CI) and geometric mean (95% CI) were 3.07 (2.64–3.50) and 1.59 (1.42–1.77) ng/mg creatinine, respectively.

Characteristics of subjects are shown in Table 1. The number of siblings was inversely associated with total difficulty scores. Both low and high birthweights were associated with higher total difficulty scores. Bottle-fed children had higher total difficulty scores. Mother's depression was associated with higher total difficulty scores of children.

Table 2 shows the association between current exposure to SHS and urinary cotinine concentrations. Forty-seven percent of children were exposed to household smoking. The sum of cigarettes parents currently smoked and the number of current smokers among cohabiters were positively associated with urinary cotinine concentrations (both *p* < 0.001).

The number of cigarettes parents currently smoked (trend *p*=0.009) and the number of current smokers among cohabiters (trend *p*=0.007) were associated with higher total difficulty scores among children (Table 3). More pack-years of exposures to parents' smoking (trend *p*=0.004) and household smoking (trend *p*=0.024) were associated with higher total difficulty scores among children. Children with higher urinary cotinine concentrations had higher total difficulty scores (trend *p*=0.005). Urinary cotinine concentrations were positively associated with hyperactivity scores (trend *p* < 0.001).

Eighty children had total difficulty scores of 14 or more (Table 4). The number of cigarettes parents currently smoked, the number of current smokers among cohabiters, the pack-years of exposures to parents' and household smoking, and urinary cotinine concentrations were associated with higher risks of behavioral problems.

Additional adjustments for the day of the week and the time of day for urine sample collection did not alter the results. For example, total difficulty scores were 8.78, 9.05, and 10.50, respectively, for the low, middle, and high tertiles of urinary cotinine concentrations (*p*=0.005, trend *p*=0.009). In addition, we reanalyzed after additional adjustment for parental history of asthma or eczema, and the results were similar; total difficulty scores were 8.69, 9.07, and 10.57, respectively, for the low, middle, and high tertiles of urinary cotinine concentrations (*p*=0.001, trend *p*=0.004).

We conducted the same analyses stratified by sex (231 boys and 206 girls). The positive associations between the indices of SHS exposures and SDQ scores were substantially not altered in boys or girls, although the associations were attenuated to be nonsignificant in girls. For example, total difficulty scores were 8.77, 9.59, and 10.95, respectively, for the low, middle, and high tertiles of urinary cotinine concentrations in boys (*p*=0.022, trend *p*=0.005), and 8.63, 8.96, and 9.64, respectively, for those in girls (*p*=0.42, trend *p*=0.97).

## 4. Discussion

Our study demonstrated positive associations between current and cumulative reported exposure to SHS in the home and behavioral problems among children. Higher urinary cotinine concentrations were also associated with more behavioral problems. These concordant findings with dose-response relationships by using various measurements for SHS exposures suggested that SHS exposure may be associated with behavioral problems in childhood.

Since the present study was cross-sectional, it is not possible to determine the causality of the association between SHS exposure and behavioral problems. However, there is the biological plausibility of the effects of SHS exposure on child behavior. Environmental tobacco smoke in experimental animals has been reported to damage cells and neuronal projection [29], upregulate nicotinic cholinergic receptor [30], and alter serotonin synaptic protein in the brain [31], possibly causing toxic effects on the developing brain. In addition, several longitudinal studies offer support for a cause-effect relationship [12, 13]. Weitzman et al. reported higher scores of Behavior Problem Index in children whose mothers only postnatally smoked a pack or more per day, compared with children whose mothers did not smoke pre- or postnatally (*p* < 0.001), among 2,256 US children aged 4–11 years [12]. In 5,991 German children, relative risks of abnormal total difficulty scores of the SDQ at 10 years of age were 2.0 (95% CI, 1.4–3.1) among children who were both pre- and postnatally exposed to parental smoking and 1.3 (95% CI, 0.9–1.9) among children only postnatally exposed, compared with children never exposed [13].

So far, a few cross-sectional studies have reported the association between objectively measured SHS exposure and behavioral problems in children [17–21]. Two studies among children with asthma have reported increased behavioral problems related to higher serum [20] or salivary [21] cotinine concentrations. In a population-based study, Hamer et al. reported that children in the highest quartile of salivary cotinine concentrations had significantly higher total difficulty score on the SDQ compared with those in the lowest quartile among healthy Scottish children aged 4–12 years [17]. High serum cotinine concentrations were associated with an increased relative risk of conduct disorder of DSM-IV criteria in US children aged 8–15 years [18]. Meanwhile, serum cotinine concentrations were not significantly associated with attention-deficit hyperactivity disorder in US children aged 4–15 years [19]. In our study, preschool children with higher urinary cotinine concentrations had significantly higher total difficulty scores on the SDQ.

One merit of this study was the assessment of SHS exposure using both detailed parent reports about passive smoking in the home and the measurement of urinary cotinine. A good participation rate and information on several potential confounders are added strengths. Although reports of SDQ can be biased based on the parent's outlook, the associations between SHS exposure and SDQ scores remained after controlling for mother's characteristics such as age of mother at delivery, mother's education years, and mother's depression.

One limitation of this study was no information on active and passive smoking of mother during pregnancy. So, we could not discriminate the effects of postnatal SHS exposure from prenatal exposure on behavioral problems. However, because the mothers smoked postnatally may have smoked during pregnancy too, we reanalyzed after excluding the mothers who currently smoked. The results were substantially unchanged. For example, total difficulty scores were 8.65, 8.94, and 10.20, respectively, for the low, middle, and high tertiles of urinary cotinine concentrations (*p*=0.023, trend *p*=0.030).

Small sample size and heights, weights, and birthweight reported by the parents were also weak points. However, we supplementarily obtained the measurement values of the height and weight from only 108 children among our subjects. The intraclass correlation coefficients between measured and parent-reported data were 0.90, 0.96, and 0.77 for height, weight, and body mass index, respectively. The mother's recalls of baby's birthweight had been reported to show high levels of agreement with registration data [32]. Therefore, these differences would not have greatly changed the associations observed in our study. In addition, although SDQ generally screens the children who may need any support or follow-up for their development, it could diagnose neither behavior nor mental disorder. Finally, the generalized application of our study might be limited by the fact that our subjects were ethnically homogeneous Japanese children with the relatively narrow range of age.

## 5. Conclusion

SHS exposure, comprehensively assessed by a detailed questionnaire and urinary cotinine measurements, was positively associated with child's behavior problems with dose-response relationships among preschool Japanese children. The importance of reducing the exposure of children to tobacco smoke, particularly in the home, was further emphasized for the prevention of behavioral problems in childhood.

## Figures and Tables

**Table 1:** Characteristics of 437 preschool children.

	*n*	Percent (%)	Total difficulty score		
			Mean	Standard error	*p* ^c^
Sex					
Boys	231	52.9	9.76^a^	0.30	0.12
Girls	206	47.1	9.07^a^	0.32	

Age					
≤4.5 y	133	30.4	9.62^a^	0.40	0.68
>4.5 to 5.5 y	124	28.4	9.56^a^	0.41	
>5.5 y	180	41.2	9.21^a^	0.34	

Body mass index (tertiles)					
Low (<14.6)	148	33.9	9.70^b^	0.38	0.58
Middle (14.6–15.7)	143	32.7	9.14^b^	0.38	
High (>15.7)	143	32.7	9.46^b^	0.38	
Missing	3	0.7			

Siblings					
None (only child)	98	22.4	10.52^b^	0.46	0.009
1st	161	36.8	9.50^b^	0.36	
2nd	177	40.5	8.76^b^	0.34	
Missing	1	0.2			

Birthweight					
2500 g or less	35	8.0	11.26^b^	0.77	0.017
>2500 to 4000 g	399	91.3	9.25^b^	0.23	
More than 4000 g	3	0.7	13.08^b^	2.62	

Feeding until 3 months old					
Breast-fed	173	39.6	8.89^b^	0.34	0.006
Mix-fed	196	44.9	9.38^b^	0.32	
Bottle-fed	68	15.6	10.97^b^	0.55	

Age of mother at delivery					
≤25 y	47	10.8	10.37^b^	0.67	0.17
26–30 y	225	51.5	9.67^b^	0.30	
31–35 y	146	37.4	8.83^b^	0.38	
≥36 y	18	4.1	9.08^b^	1.08	
Missing	1	0.2			

Mother's education (years)					
≤12 y	138	31.6	9.58^b^	0.39	0.51
13–15 y	209	47.8	9.53^b^	0.32	
≥16 y	75	17.2	8.88^b^	0.53	
Missing	15	3.4			

Mother's depression					
No	342	78.3	9.26^b^	0.25	0.018
Yes	60	13.7	10.79^b^	0.59	
Missing	35	8.0			

^a^Crude mean, ^b^sex- and age-adjusted mean, and ^c^analysis of variance or analysis of covariance. The total difficulty score was assessed by the Strengths and Difficulties Questionnaire.

**Table 2:** Associations between current exposure to tobacco smoke and urinary cotinine concentrations.

	*n*	Percent (%)	Urinary cotinine levels (ng/mg creatinine)		*p* ^a^
			Geometric mean	95% CI	
Number of cigarettes parents currently smoked (cigarettes per day)					
0	241	55.1	0.99	(0.15–6.49)	<0.001
>0 to 20	152	34.8	2.30	(0.25–20.93)	
>20	44	10.1	5.88	(1.10–31.62)	

Number of current smokers among cohabiters (persons)					
0	233	53.3	0.94	(0.15–5.78)	<0.001
1	149	34.1	2.09	(0.26–16.68)	
≥2	55	12.6	6.79	(1.26–36.70)	

CI: confidence interval. ^a^By analysis of variance.

**Table 3:** Means of SDQ scores^a^ according to exposure to tobacco smoke among preschool children.

	*n*	Total difficulty score	Peer problems	Hyperactivity	Conduct problems	Emotional symptoms	Prosocial behavior
Number of cigarettes parents currently smoked (cigarettes per day)							
0	238	9.06	1.63	3.41	2.13	1.89	6.21
>0 to 20	151	9.52	1.85	3.52	2.17	1.98	5.85
>20	44	11.20	1.90	3.99	2.65	2.66	5.96
*p*		0.020	0.29	0.25	0.11	0.056	0.24
Trend *p*		0.009	0.14	0.13	0.085	0.039	0.18

Number of current smokers among cohabiters (persons)							
0	230	8.93	1.59	3.37	2.11	1.86	6.19
1	148	9.75	1.86	3.63	2.17	2.08	5.71
≥2	55	10.74	2.00	3.77	2.61	2.35	6.49
*p*		0.026	0.091	0.31	0.089	0.23	0.024
Trend *p*		0.007	0.032	0.13	0.066	0.086	0.86

Cumulative exposure to parents' smoking (range, pack-years)							
0	204	8.89	1.58	3.29	2.11	1.91	6.25
Low (0.07–4.34)	112	9.52	1.75	3.62	2.13	2.02	5.85
High (4.36–14.48)	112	10.48	2.01	3.82	2.47	2.17	5.94
*p*		0.017	0.053	0.091	0.11	0.53	0.21
Trend *p*		0.004	0.016	0.031	0.059	0.26	0.18

Cumulative exposure to household smoking (range, pack-years)							
0	197	8.92	1.56	3.30	2.12	1.94	6.22
Low (0.05–4.47)	114	9.70	1.76	3.73	2.15	2.06	5.98
High (4.48–24.28)	115	10.17	1.99	3.70	2.42	2.06	5.89
*p*		0.070	0.053	0.14	0.24	0.82	0.37
Trend *p*		0.024	0.015	0.099	0.12	0.60	0.18

Urinary cotinine levels (range, ng/mg creatinine)							
Low (0.03–0.95)	145	8.72	1.55	3.16	2.12	1.89	6.32
Middle (0.96–2.30)	144	9.09	1.64	3.37	2.11	1.97	5.86
High (2.31–39.7)	144	10.52	2.02	4.00	2.37	2.13	6.01
*p*		0.002	0.015	0.002	0.27	0.58	0.15
Trend *p*		0.005	0.12	<0.001	0.19	0.61	0.73

^a^By analysis of covariance after adjustments for rater (mother, father), sex, age, body mass index, siblings (none, 1st, and 2nd), birthweight, feeding until 3 months old (breast-, mix-, and bottle-fed), age of mother at delivery, mother's education (≤12, 13–15, ≥16 y), and mother's depression (yes, no). SDQ: the Strengths and Difficulties Questionnaire; CI: confidence interval.

**Table 4:** Odds ratios (ORs) of behavioral problems according to exposure to tobacco smoke among preschool children.

	Behavioral problems					
	(−)	(+)	Crude OR	(95% CI)	Adjusted OR^a^	(95% CI)
Number of cigarettes parents currently smoked (cigarettes per day)						
0	205	36	1.00	(Ref)	1.00	(Ref)
>0 to 20	123	29	1.34	(0.78–2.30)	1.43	(0.79–2.58)
>20	29	15	2.95	(1.44–6.03)	3.29	(1.48–7.34)
Trend *p*			0.005		0.005	
Number of current smokers among cohabiters (persons)						
0	200	33	1.00	(Ref)	1.00	(Ref)
1	118	31	1.59	(0.93–2.73)	1.81	(1.00–3.25)
≥2	39	16	2.49	(1.25–4.95)	2.64	(1.20–5.82)
Trend *p*			0.007		0.009	
Cumulative exposure to parents' smoking (range, pack-years)						
0	179	26	1.00	(Ref)	1.00	(Ref)
Low (0.07–4.34)	89	25	1.93	(1.06–3.54)	1.76	(0.92–3.40)
High (4.36–14.48)	85	28	2.27	(1.25–4.10)	2.67	(1.38–5.16)
Trend *p*			0.006		0.003	
Cumulative exposure to household smoking (range, pack-years)						
0	172	26	1.00	(Ref)	1.00	(Ref)
Low (0.05–4.47)	90	26	1.91	(1.05–3.48)	1.76	(0.92–3.39)
High (4.48–24.28)	90	26	1.91	(1.05–3.48)	2.17	(1.10–4.28)
Trend *p*			0.032		0.025	
Urinary cotinine levels (range, ng/mg creatinine)						
Low (0.03–0.95)	127	19	1.00	(Ref)	1.00	(Ref)
Middle (0.96–2.30)	123	23	1.25	(0.65–2.41)	1.17	(0.59–2.33)
High (2.31–39.7)	107	38	2.37	(1.29–4.36)	2.22	(1.16–4.24)
Trend *p*			0.017		0.041	

^a^Adjustments for rater (mother, father), sex, age, body mass index, siblings (none, 1st, and 2nd), birthweight, feeding until 3 months old (breast-, mix-, and bottle-fed), age of mother at delivery, mother's education (≤12, 13–15, ≥16 y), and mother's depression (yes, no). CI: confidence interval. Behavioral problem was defined as ≥14 of total difficulty score.

## Data Availability

The data used to support the findings of this study have not been made available due to confidentiality.
